# Cyanoarylporphyrazines with High Viscosity Sensitivity: A Step towards Dosimetry-Assisted Photodynamic Cancer Treatment

**DOI:** 10.3390/molecules26195816

**Published:** 2021-09-25

**Authors:** Irina V. Balalaeva, Tatiana A. Mishchenko, Victoria D. Turubanova, Nina N. Peskova, Natalia Y. Shilyagina, Vladimir I. Plekhanov, Svetlana A. Lermontova, Larisa G. Klapshina, Maria V. Vedunova, Dmitri V. Krysko

**Affiliations:** 1 Department of Biophysics, Institute of Biology and Biomedicine, Lobachevsky State University of Nizhny Novgorod, 23 Gagarin ave., 603022 Nizhny Novgorod, Russia; irin-b@mail.ru (I.V.B.); nin-22@yandex.ru (N.N.P.); nat-lekanova@yandex.ru (N.Y.S.); 2 Department of Neurotechnology, Institute of Biology and Biomedicine, Lobachevsky State University of Nizhny Novgorod, 23 Gagarin ave., 603022 Nizhny Novgorod, Russia; saHarnova87@mail.ru (T.A.M.); vikaturu@mail.ru (V.D.T.); MVedunova@yandex.ru (M.V.V.); 3 Department of Basic and Medical Genetics, Institute of Biology and Biomedicine, Lobachevsky State University of Nizhny Novgorod, 23 Gagarin ave., 603022 Nizhny Novgorod, Russia; 4 Department of Radiophysical Methods in Medicine, Federal Research Center Institute of Applied Physics of the Russian Academy of Sciences, 46 Ul’yanov Street, 603950 Nizhny Novgorod, Russia; Plehanov_VI@mail.ru; 5 Sector of Chromophors for Medicine, G.A. Razuvaev Institute of Organometallic Chemistry of the Russian Academy of Sciences, 49 Tropinin st., 603137 Nizhny Novgorod, Russia; lermontovasa@rambler.ru (S.A.L.); klarisa@ioms.ras.ru (L.G.K.); 6 Cell Death Investigation and Therapy Laboratory (CDIT), Anatomy and Embryology Unit, Department of Human Structure and Repair, Ghent University, C. Heymanslaan 10, Building B3, 4th Floor, 9000 Ghent, Belgium; 7 Cancer Research Institute Ghent, 9000 Ghent, Belgium; 8 Department of Pathophysiology, Sechenov First Moscow State Medical University (Sechenov University), 8-2 Trubetskaya st., 119991 Moscow, Russia

**Keywords:** photodynamic therapy, cancer treatment, photosensitizers, porphyrazines, intracellular viscosity, fluorescent molecular rotors, cell death

## Abstract

Despite the significant relevance of photodynamic therapy (PDT) as an efficient strategy for primary and adjuvant anticancer treatment, several challenges compromise its efficiency. In order to develop an “ideal photosensitizer” and the requirements applied to photosensitizers for PDT, there is still a need for new photodynamic agents with improved photophysical and photobiological properties. In this study, we performed a detailed characterization of two tetracyanotetra(aryl)porphyrazine dyes with 4-biphenyl (**pz II**) and 4-diethylaminophenyl (**pz IV**) groups in the periphery of the porphyrazine macrocycle. Photophysical properties, namely, fluorescence quantum yield and lifetime of both photosensitizers, demonstrate extremely high dependence on the viscosity of the environment, which enables them to be used as viscosity sensors. **Pz** **II** and **pz IV** easily enter cancer cells and efficiently induce cell death under light irradiation. Using fluorescence lifetime imaging microscopy, we demonstrated the possibility of assessing local intracellular viscosity and visualizing viscosity changes driven by PDT treatment with the compounds. Thus, **pz II** and **pz IV** combine the features of potent photodynamic agents and viscosity sensors. These data suggest that the unique properties of the compounds provide a tool for PDT dosimetry and tailoring the PDT treatment regimen to the individual characteristics of each patient.

## 1. Introduction

The past decade has witnessed major breakthroughs in photodynamic therapy (PDT) as an efficient strategy for primary and adjuvant anticancer treatment. PDT is a clinically approved minimally invasive procedure based on a photodynamic agent’s local or systemic administration (photosensitizer) with subsequent selective accumulation in tumor tissue and excitation by irradiation with visible light of appropriate wavelength. The excited photosensitizer reacts with cellular oxygen, resulting in the generation of cytotoxic reactive oxygen species (e.g., singlet oxygen (^1^O_2_), superoxide anion (O_2_^−•^), hydroperoxide radical (HOO^•^), peroxides (H_2_O_2_, ROOH), and hydroxyl radical (HO^•^)), and initiating molecular mechanisms which lead to tumor tissue destruction and vascular damage [1,2,3]. The efficient development of photodynamic reaction depends primarily on the photophysical and photochemical properties of the photosensitizer [4,5,6], its tissue distribution [7,8], its uptake by cancer cells and subcellular localization [4,9,10,11], and the irradiation dose [12,13].

Despite significant progress in PDT, there are still several challenges that compromise its efficiency. For instance, oxygen deficiency (hypoxia) in the tumor microenvironment can significantly diminish PDT efficiency for solid tumors [1,14,15]. Insufficient selectivity of the photosensitizer to the tumor and its accumulation in normal organs and tissues, especially skin and eyes, lead to undesirable photoinduced side effects [8,16,17]. Moreover, several types of tumors often become resistant to apoptotic and necroptotic cell death modalities, making their induction by PDT no longer an option [18,19]. Therefore, there is still a need for new photosensitizers with improved photodynamic properties and less cytotoxic effects for normal tissues.

We recently reported the novel group of tetracyanotetra(aryl)porphyrazines (hereinafter, **pz**) as prospective photosensitizers for PDT [20,21,22,23,24]. The structure of **pz** determines their unique photophysical properties; namely, an ability to induce the production of strongly cytotoxic singlet oxygen combined with high viscosity sensitivity of the fluorescence parameters [20]. This can be explained by the fact that **pz** belongs to the molecular rotors: excitation of this type of molecule due to light absorption leads to charge transfer followed by intramolecular rotation. This results in a high rate of non-radiative relaxation of the excited state of the molecular rotor. In viscous media, the rotating is inhibited, resulting in a drastic increase in fluorescence intensity and fluorescence lifetime. We should underline that modification of the peripheral aryl groups coupled with a macrocycle core can significantly affect the photophysical and cytotoxic properties of **pz**, thus opening new prospects for fine-tuning of **pz** as an effective tool for PDT treatment [21,22,23,24].

Moreover, in our previous study, we showed that several compounds of **pz** group (**pz I–IV**) can be considered as candidate photosensitizers for PDT treatment of glioma [25]. Of note, the special requirement for brain tumors is the minimal impact of photosensitizers on neurons functioning. The lowest neuronal toxicity was obtained for **pz** with four 4-biphenyl groups in the aryl frame of the macrocycle (**pz II**). Mild toxicity was detected for compound with 4-diethylaminophenyl groups (**pz IV**).

In this study, we performed a detailed characterization of **pz II** and **pz IV** in terms of photophysical and photobiological properties, aiming at their future PDT application. Herein, we provided data on photophysical properties of photosensitizers, their viscosity sensitivity, as well as accumulation dynamics and subcellular distribution in cancer cells. Finally, we performed a comparative analysis of the photodynamic properties of **pz II** and **pz IV** for two cancer cell lines of different origin (murine glioma GL261 and human epidermoid carcinoma A431). Using fluorescence lifetime imaging microscopy (FLIM), we demonstrated the possibility of assessing local intracellular viscosity using **pz II** and **pz IV** as viscosity sensors and tracking the viscosity changes driven by PDT treatment. We assume that measuring the fluorescence lifetime of the tested compounds reflects the physiological states of the cells and can provide a tool for PDT dosimetry and tailor the PDT treatment regimen to the individual characteristics of each patient.

## 2. Results

### 2.1. Photophysical Properties of pz II and pz IV

**Figure 1:**
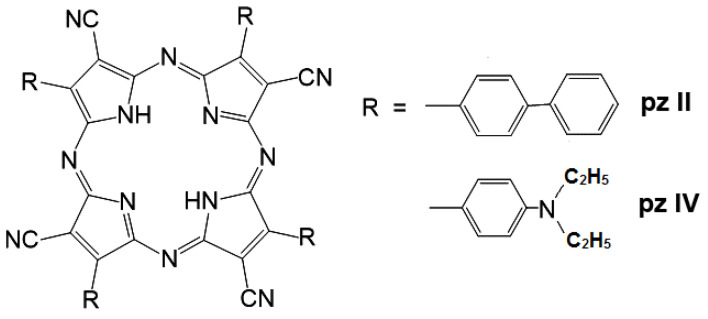
A chemical structure of the synthesized cyanoarylporphyrazines: **pz II** and **pz IV**.

Two tetracyanotetra(aryl)porphyrazine dyes were tested with 4-biphenyl (**pz II**) and 4-diethylaminophenyl (**pz IV**) groups in the periphery of the porphyrazine macrocycle. For the synthesis of **pz II** and **pz IV**, we used a synthetic approach using a metal template assembly of the porphyrazine frameworks that we developed and described in detail previously [20,21,26]. The chemical structure of the dyes is shown in Figure 1.

Both of the compounds demonstrated intensive light absorption, with a maximum in the red spectral region at 592–594 nm (Q-band) and a molar extinction coefficient exceeding 10^4^ (Table 1). In a water solution, the fluorescence of both of the compounds was rather weak, with quantum yields of about 0.001–0.002. The fluorescence peak of **pz II** has a slight bathochromic shift compared to **pz IV** due to the extension of the π-conjugated aromatic system (Table 1).

**Figure 2:**
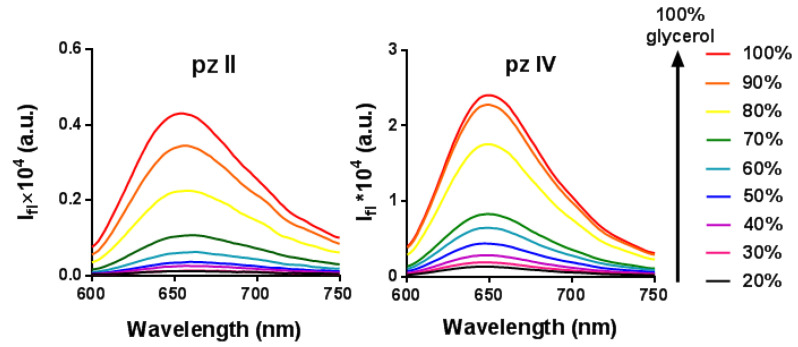
Enhancement of the fluorescence intensity of **pz II** and **pz IV** in ethanol–glycerol solutions at room temperature.

To investigate whether the photophysical properties of **pz II** and **pz IV** depend on viscosity, we analyzed their fluorescence spectra in ethanol–glycerol solutions with a glycerol volume concentration from 20 to 100%. The viscosities of the corresponding solutions at room temperature varied from 3.9 to 820 cP. No broadening or spectral shift was observed (Figure 2), which would indicate aggregation. However, fluorescence intensity (i.e., fluorescence quantum yield) demonstrated an increase of more than two orders of magnitude in viscous media; the quantum yield values in glycerol were 0.23 and 0.60 for **pz II** and **pz IV,** respectively.

**Figure 3:**
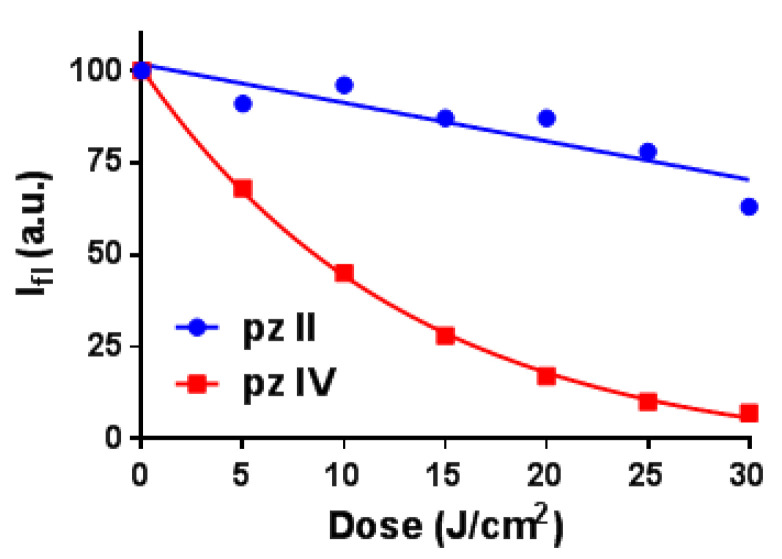
Photobleaching of **pz II** and **pz IV** in ethanol–glycerol solutions with a viscosity of 30 cP induced by irradiation with power density 20 mW/cm^2^.

Note that there is a difference in the photostability of the two compounds (Figure 3 and Figure S1 from Supplementary Materials). While **pz IV** in media with a viscosity of 30 cP demonstrated rapid photobleaching under irradiation with power density of 20 mW/cm^2^, **pz II** was much more stable, assuming a higher contribution of excited state relaxation through intramolecular rotation.

The fluorescence quantum yield’s *φ* dependence on the medium viscosity *η* followed the well-known Förster–Hoffmann equation for fluorescent molecular rotors [27]:

(1) lg(φ)=z+αlg(η)

where z and α are constants.

**Figure 4:**
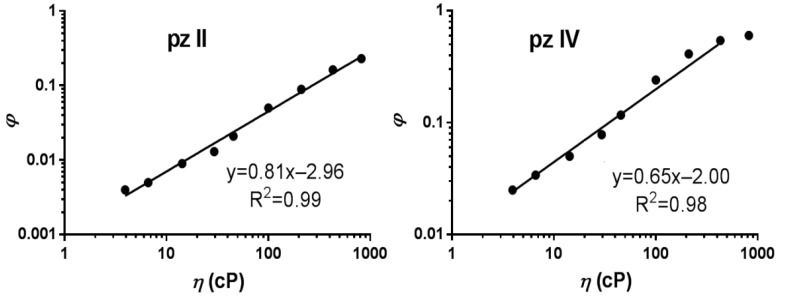
Dependence of the fluorescence quantum yield (*φ*) of **pz II** and **pz IV** on the viscosity (*η*) measured in ethanol–glycerol solutions. The data followed the Förster–Hoffmann equation with coefficients of viscosity sensitivity of 0.81 and 0.65, respectively.

The coefficient α reflects the viscosity sensitivity and is 0.81 and 0.65 for **pz II** and **pz IV,** respectively (Figure 4). Such high viscosity dependence coefficients are characteristic of fluorescence molecular rotors [28,29].

**Figure 5:**
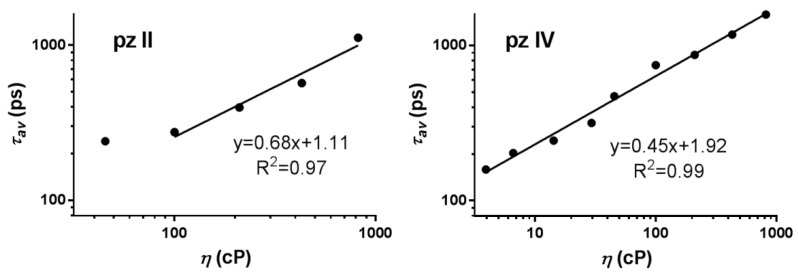
Dependence of the fluorescence lifetime of **pz II** and **pz IV** on the viscosity measured in ethanol–glycerol solutions. The data followed the Förster–Hoffmann equation with coefficients of viscosity sensitivity of 0.68 and 0.45, respectively.

The similar dependence was registered for fluorescence lifetime (Figure 5 and Figure S2 from Supplementary Materials). The averaged lifetime *τ_av_* dependence on the medium viscosity can be described as:

(2) lg(τav)=lg(zkr)+αlg(η)

where *k_r_* is the constant of radiative relaxation.

Of note, there is a difference in the properties of the two studied compounds. Due to technical limitations, we were not able to relevantly measure lifetimes lower than ~200 ps. For **pz II**, the fluorescence lifetimes were very short in media with a viscosity of up to 100 cP (Figure 5); this is in line with the lower values of quantum yield for this compound (Figure 4). Thus, we registered the viscosity dependence for **pz II** in 100–850 cP range. On the contrary, **pz IV** has a high fluorescence lifetime and quantum yield in the entirety of the analyzed viscosity range. At the same time, the coefficient of viscosity sensitivity was higher for **pz II**: 0.68 compared to 0.45 for **pz IV** when measuring fluorescence lifetime.

**Figure 6:**
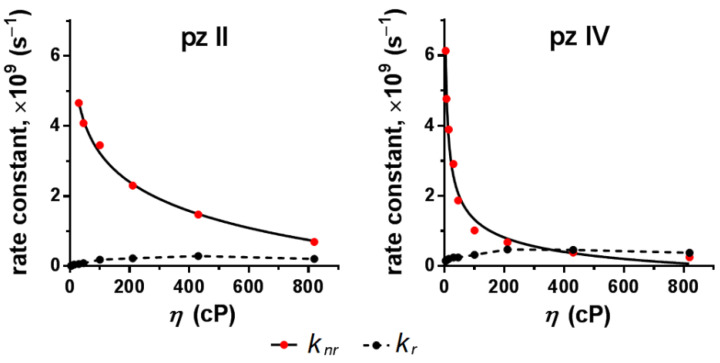
Dependence of constants of radiative (*k_r_)* and non-radiative (*k_nr_)* relaxation of **pz II** and **pz IV** on the viscosity.

Based on the performed measurements, we calculated the rate constants of radiative *k_r_* and non-radiative *k_nr_* relaxation (Figure 6). For both compounds, *k_r_* remained virtually unchanged in media with increasing viscosity; however, *k_nr_* drastically decreased. This proves the hypothesis that the rise in fluorescence quantum yield and lifetime of **pz II** and **pz IV** in viscous media resulted precisely from the limitation of non-radiative processes. The difference in the behavior of the two dyes is most clearly manifested in the rate constants’ dependence on viscosity (Figure 6): the higher constant of non-radiative relaxation for **pz II**, demonstrating its prevailing role in **pz II** relaxation in the studied viscosity range, and the higher sensitivity of **pz IV** to viscosity changes in low viscous media.

Thus, **pz II** and **pz IV** demonstrate high viscosity sensitivity in solution, presumably due to belonging to the molecular rotors; both the quantum yield and fluorescence lifetime of the compounds can be measured for viscosity sensing purposes.

### 2.2. Cellular Uptake and Photodynamic Properties of pz II and pz IV

**Figure 7:**
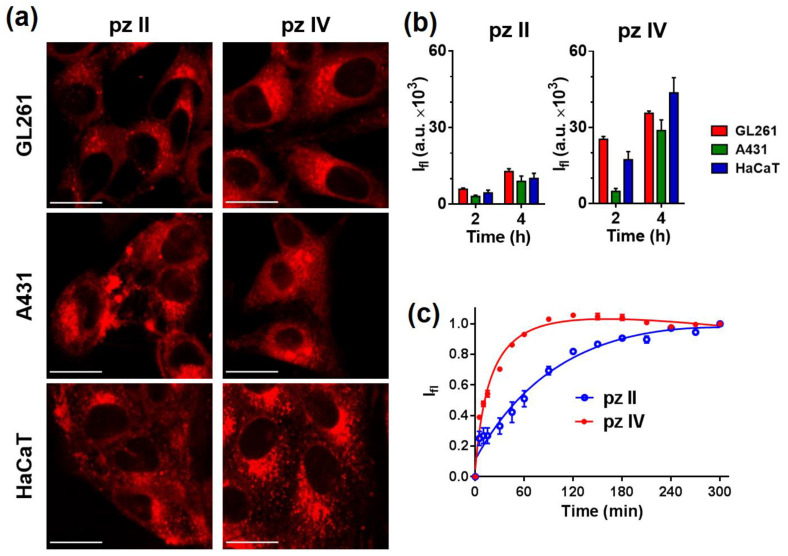
Analysis of the cellular uptake of **pz II** and **pz IV**. (**a**) Representative confocal images of murine glioma GL261, human epidermoid carcinoma A431, and human keratinocytes HaCaT cells after 240 min of incubation with the **pz** (10 µM). Scale bar, 20 μm. (**b**) Relative accumulation of the **pz** by cells of different lines after 2 and 4 h incubation, estimated from confocal microscopy experiment (n ≥ 10). (**c**) Dynamics of **pz II** and **pz IV’s** accumulation in A431 cells. The **pz** was added to the culture medium at a concentration of 5 μM, and a plate reader repeatedly measured the fluorescence in the well of the plate with the cell culture. The signal growth results from **pz** entering the intracellular environment with a higher local viscosity compared to outer culture medium.

To test the photobiological properties of **pz II** and **pz IV**, we used two cancer cell lines, murine glioma GL261 and human epidermoid carcinoma A431, and a line of immortalized human keratinocytes HaCaT. The choice of cancer lines was determined by the cells’ origin from the tumors that are acceptable for PDT treatment. Both compounds were taken up by the cells; however, the rate of cellular uptake is significantly differed (Figure 7). Comparing the cell lines, the most intensive fluorescence signal in confocal images was obtained in glioma GL261 cells; the least intensive was found in carcinoma A431 cells.

It should be noted that the unique photophysical properties of **pz** allowed us to apply an original measuring technique using continuous registering of the fluorescence of the cell culture in a multiwall plate with a plate reader. **pz II** and **pz IV** were added in the incubation media; virtually no fluorescence signal was detected in the media itself due to its low viscosity. When entering the cell, the **pz** enters a much more viscous environment, leading to significant enhancement of the detected fluorescence signal. The molecule of **pz II** with larger aryl radicals resulted in relatively slow uptake and reaching maximal signal in about 3–4 h (Figure 7b,c). In the case of **pz IV**, 1.5 h was enough to reach the saturation level of the fluorescence.

**Figure 8:**
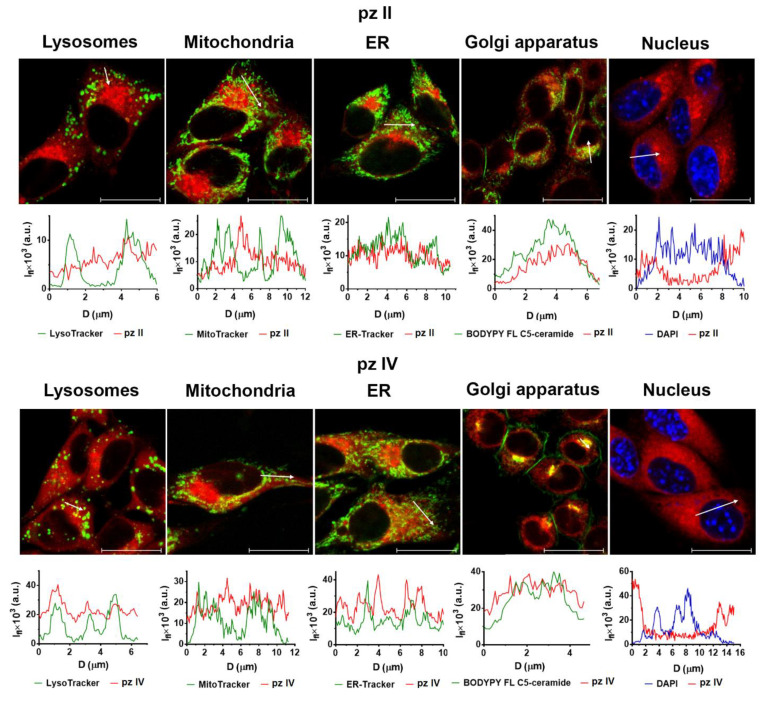
A subcellular distribution of **pz II** and **pz IV** in the glioma GL261 cells. Glioma cells were incubated with 10 µM **pz** for 4 h. The following dyes were used for staining of the organelles: LysoTracker Green for lysosomes; MitoTracker Green for mitochondria; ER-Tracker for ER; DAPI for nucleus. The plots present the profiles of the fluorescence signal (I_fl_) of the **pz** (red line) and organelle dye (green and blue line) along the white arrow indicated in the corresponding image. Scale bar, 20 μm.

Predominant **pz II** and **pz IV** localize in both glioma GL261 and carcinoma A431 cells in the endoplasmic reticulum (ER) and Golgi apparatus (Figure 8). This is in accordance with the amphiphilic properties of the compounds. Of note, we previously reported that in murine neurons, both dyes were mostly held in vesicles with partial localization of **pz II** in ER [25]. Thus, the origin and properties of the cancer cells can influence the intracellular distribution of **pz**.

**Figure 9:**
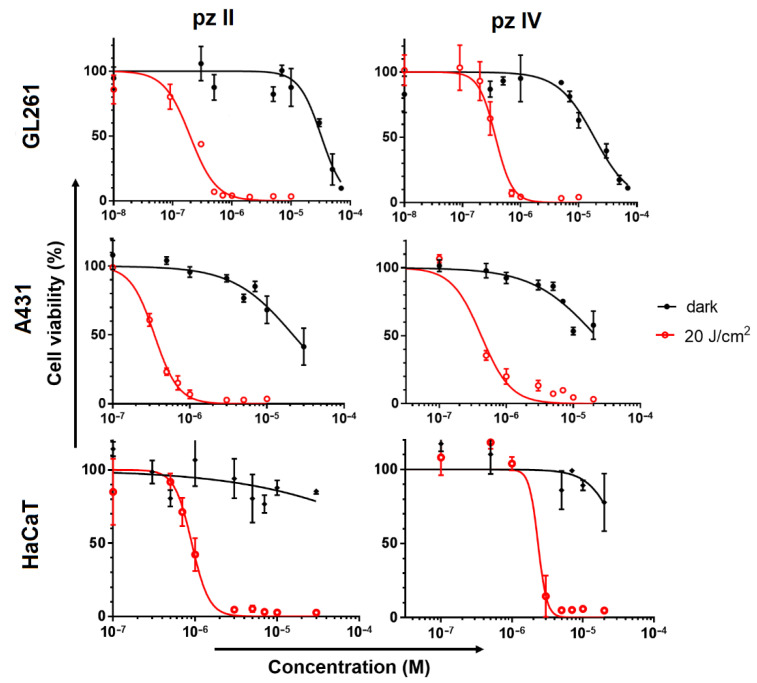
Dark toxicity and photodynamic activity of **pz II** and **pz IV** against murine glioma GL261, human epidermoid carcinoma A431 and human keratinocytes HaCaT cells. The cells were incubated with **pz** in serum-free medium for 4 h and then exposed to light irradiation (λ_ex_ 615–635 nm, 20 J/cm^2^) or stayed in the dark for the same time interval. After 24 h, the cell viability was analyzed by MTT assay. The representative dose–response curves from three independent experiments (each in triplicate) are shown. The data represent the mean values ± SD (n = 3).

Exposure to **pz II** and **pz IV** in the dark led to a reduction in the viability of cancer cells only in concentrations of about 2 × 10^−5^ M. Under light irradiation, both compounds demonstrated light dose-dependent photodynamic activity (Figure S3 from Supplementary Materials). Under a dose of 20 J/cm^2^, this was manifested in a significant (>50%) loss of cancer cell viability when they were treated with sub-micromolar **pz** concentrations (Figure 9). Glioma cells are shown to be relatively more sensitive to the **pz**-based PDT treatment: IC_50_ values towards GL261 were 0.19 and 0.37 µM for **pz II** and **pz IV**; in the case of carcinoma A431 cells, the values were 0.35 and 0.42 µM, respectively. The photodynamic index (the ratio of IC_50_ in the dark and under irradiation) was more than 50, which is an excellent reason to call **pz II** and **pz IV** potent photosensitizers.

Of interest, the photoinduced toxicity of **pz II** and **pz IV** against normal keratinocytes was significantly lower than against cancerous skin cells, contrary to the relative **pz** accumulation shown above: IC_50_ values towards HaCaT were 0.9 and 2.3 µM, respectively. These values indicate that there is no strict correlation between pz accumulation and its cytotoxic activity. The peculiarities of redox metabolism and antioxidant pool in cancer cells can result in their high sensitivity to PDT treatment.

### 2.3. Intracellular Viscosity Sensing during PDT Treatment with pz II and pz IV

The most intriguing question was whether we can detect intracellular viscosity using **pz II** and **pz IV** sensor properties and if we can register PDT-induced viscosity changes. We performed a series of experiments using fluorescence lifetime imaging microscopy (FLIM) to answer this question. The fluorescence lifetime of **pz** was estimated in intact cells; then, one-half of the microscopic field was treated with an intense laser to induce PDT response. The FLIM images of the whole field were obtained immediately after and at different time points after PDT. The described approach allowed us to take into account unavoidable cell irradiation during the imaging procedure; the cells in the non-treated area serve as a control for analysis of the results.

**Figure 10:**
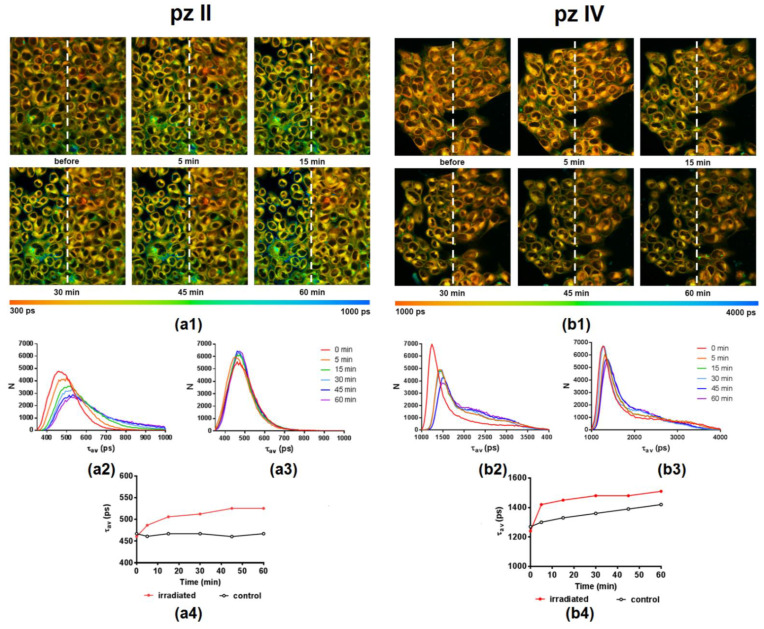
Representative FLIM images illustrating **pz II’s** (**a1**) and **pz IV’s** (**b1**) fluorescence lifetime (τ_av_) distribution in human epidermoid carcinoma A431 cells before and at different time points after PDT treatment. Photodynamic exposure (594 nm, 20 J/cm^2^) was performed in the left half of the field of view; irradiated and control areas are divided by a white dotted line. λ_ex_, 800 nm, λ_em_, 652–740 nm. The color scale range is between 300 and 1000 ps for **pz II** and 1000 and 4000 ps for **pz IV**. Image size, 212 × 212 μm. The distribution histograms of a fluorescence lifetime (τ_av_) in irradiated and control regions for **pz II** (**a2**,**a3**) and **pz IV** (**b2**,**b3**) and the plot of τ_av_ vs. time after irradiation (**a4**,**b4**) are shown.

**pz II** and **pz IV** demonstrated a fluorescence lifetime distribution in cells with maxima at ~470 and ~1250 ps (Figure 10). It is necessary to underline that the intracellular environment fundamentally differs from a glycerol–ethanol solution; therefore, it is not relevant to apply the described above results to calculate intracellular viscosity. The hydrophobicity of the local microenvironment and the interactions of **pz** with intracellular proteins, lipids, nucleic acids, and supramolecular complexes are of the utmost importance. In any case, the registered fluorescence lifetimes evidence the strong hindrance of intramolecular movement in both compounds.

PDT treatment induced a shift towards longer **pz** lifetimes in the irradiated area. This starts immediately after a short period of irradiation and continues during 1 h of observation (Figure 9). In the case of **pz II**, this shift was observed from 470 to 525 ps; for **pz IV**, this shift was from 1250 to 1500 ps. In the latter case, we should note the pronounced changes in the control area in the shown example, presumably due to the cell irradiation during the imaging procedure. Such changes or their absence were detected randomly and seemingly depended on a moderate variation in the initial physiological state of the cell culture. Thus, the viscosity sensitivity of **pz** fluorescence provided us with a tool to visualize changes in intracellular viscosity, namely its increase, induced by PDT.

## 3. Discussion

Photodynamic therapy has been used in clinical practice since the middle of the 20th century; by now, it has become widely used in cancer treatment. Over several decades, a substantial amount of experimental and clinical data has been accumulated, which allowed the development of a list of requirements for the photosensitizers used. Among them, the most significant are: (1) photosensitizers should have a constant chemical composition and simple synthesis; (2) they should accumulate quickly and selectively in tumor cells, providing a stable and high quantum yield of singlet oxygen in PDT with subsequent induction of cell death; (3) they should have a minimal cytotoxic effect on normal (non-transformed) cells [30,31,32]. Despite significant efforts, no “ideal” photosensitizers have been created to date, and plenty of novel photoactive dyes are tested as potential photosensitizers every year. Moreover, new findings in the mechanisms of tumor eradication during PDT, tumor immunity, and overall drug development dictate the need to expand the requirements applied to photosensitizers and revise the concept of an “ideal photosensitizer”.

In the current study, we present two tetracyanotetra(aryl)porphyrazines, **pz II** and **pz IV**, that efficiently induce photodynamic death on murine glioma GL261 and human epidermoid carcinoma A431 cells. **Pz II** demonstrates a lower rate of cellular uptake (Figure 7) but slightly higher toxicity against cancer cells (Figure 9). However, the value of the photodynamic index at more than 50 when irradiated at a dose of 20 J/cm^2^ supports the high potency of both compounds as photosensitizers.

The advantage of **pz II** and **pz IV** compared to clinically approved ”classic” photosensitizers is their sensor properties. The molecular rotors’ photophysical properties of **pz II** and **pz IV** drastically change depending on the viscosity of the surrounding medium. In a less viscous medium, the excitation of the **pz** molecule is followed by the intramolecular twisting or rotation of side aryl groups, resulting in a very high constant of non-radiative relaxation (Figure 6) and subsequently a low quantum yield (Figure 4) and fluorescence lifetime (Figure 5). In high-viscosity media, intramolecular rotation is inhibited. Thus, we register an increase in the emission intensity and the fluorescence lifetime by several orders of magnitude (Figure 4 and Figure 5). The sensitivity of the fluorescence lifetime is of particular interest, since it can be measured in a complex biological media when no data on the precise concentration of the sensor are available.

It has been reported that the cell response to PDT treatment is accompanied by an increase in intracellular viscosity [20,33,34]. We registered the same cell reaction using **pz II** and **pz IV** as photosensitizers and, at the same time, viscosity sensors. Higher viscosity can intensify lipid peroxidation in membranes, leading to lipid-to-lipid and lipid-to-protein cross-linking through Schiff base formation [35]. The second important factor is an accumulation of misfolded and partially denatured proteins [36]. This is seemingly the most potent factor influencing the surrounding environment of ER-located porphyrazines during the development of ER stress. In any case, viscosity changes start immediately after the beginning of the PDT treatment and indicate the physiological state of the irradiated cells.

The use of **pz** in combination with time-resolved microscopy allowed for obtaining viscosity maps (microviscosity) of cancer cells with submicron resolution and visualizing PDT-driven viscosity changes at the subcellular level (Figure 10). The idea to use cyanoarylporphyrazines to follow viscosity changes during PDT was firstly presented for fluorophenyl derivative [20]. In the present work, we described two porphyrazines with several benefits over previously published compounds: a red shift of absorption and emission maxima that is important for future in vivo application; higher PDT activity against cancer cells and large photodynamic index; and low toxicity against neuronal cells [25], which allows **pz IV** and especially **pz II** application for treatment of gliomas. We assume that real-time assessment of fluorescence lifetime of **pz II** and **pz IV** provides a possibility for light dosimetry and correction of the irradiation regimen. Thus, an absence of pronounced “viscosity” response during irradiation points toward the insufficient intensity of treatment, and vice versa, a fast and robust response indicates the substantial impairment of irradiated cells. Looking ahead, fluorescence lifetime measurement can be the rational basis for the individualization of the PDT treatment. In this case, the estimation of individual responsivity to PDT treatment can be used for light-dose optimization to reach the highest treatment efficacy without severe damage to surrounding normal tissues.

Maintaining a low toxicity effect for normal tissues in the organism is one of the essential requirements for photosensitizers. Our previous in vitro studies showed that **pz II** and **pz IV** had a light and mild cytotoxic effect, respectively, for primary neuronal cultures, which are among the most sensitive cells to any physicochemical stimuli and stress [25], which shows good promise for the future in vivo application of both of these compounds. In the present study, we have shown lower photoinduced toxicity of **pz II** and **pz IV** against normal keratinocytes compared to skin cancer cells.

A strategy of targeted PDT has been proposed to minimize the side effects mediated by the cytotoxicity to normal cells, representing third-generation photosensitizers. A photoactive chromophore is combined with a targeting moiety or vehicle (i.e., liposomal forms, nanocarriers, conjugates with sugar molecules, monoclonal antibodies, or peptides) that significantly improves the pharmacokinetics of the photosensitizer and allows the reduction in the total administered dose [1,37,38,39]. We previously demonstrated that other compounds of tetracyanotetra(aryl)porphyrazine group could be efficiently loaded into polymer brush nanoparticles [40,41]. Notably, the developed nanocarriers had less dark toxicity than the photosensitizer itself. Another example is the loading of tetracyanotetra(aryl)porphyrazines into liposomes [42]. Thus, in addition to their low toxicity, **pz II** and **pz IV** can be encapsulated in a nano-sized vehicle for improvement of the targeted delivery to the tumor tissue via an enhanced permeability and retention effect.

One of the newly established requirements for an “ideal” photosensitizer is the induction of a regulated cell death modality with immunogenic properties. This requirement is descended from uncovering the mechanisms of the interaction of dying/dead cancer cells with immune cells and establishing this interaction as a crucial factor of cancer treatment efficiency. Immunogenic cell death (ICD) has an adjuvant-like effect mediated by the release of damage-associated molecular patterns (DAMPs) responsible for the recruitment and maturation of antigen-presenting cells followed by the induction of T-cell adaptive anticancer immunity and tumor cells eradication [1,43,44,45]. The ability of photosensitizers to induce ICD is closely linked with their localization in cells [1,45]. **Pz II** and **pz IV** accumulate in ER (Figure 8), which might indicate their ability to induce ER-stress during PDT, one of the main prerequisites of ICD induction [9,45]. The power of these particular compounds to induce ICD needs to be further investigated; however, we can now assert that tetracyanotetra(aryl)porphyrazines fulfill this requirement. In our previous study, we showed that cancer cells subjected to PDT with two compounds of cyanoarylporphyrazine group with 9-phenanthrenyl and 4-(4-fluorobenzyoxy)phenyl substituted in the aryl frame of the macrocycle demonstrate typical features of ICD: release of crucial DAMPs (e.g., ATP and HMGB1), which induce activation and maturation of dendritic cells in vitro [11]. Moreover, cancer cells stimulated with pz-PDT served as a potent vaccine in a prophylactic tumor vaccination model in vivo, activating the adaptive immune system, thereby ensuring effective protection of mice from tumor growth after challenge with the viable cancer cells.

To sum up, our studies reveal that **pz II** and **pz IV** could be considered as potent agents for PDT with high viscosity sensitivity of their photophysical properties. The latter benefit feature allowed for visualizing the PDT-driven rise in intracellular viscosity. We assumed that the unique properties of **pz II** and **pz IV** provide the basis for dosimetry-assisted photodynamic cancer treatment.

## 4. Materials and Methods

### 4.1. Analysis of Spectral Properties and Fluorescence Quantum Yield

The absorption and fluorescence spectra of **pz II** and **pz IV** were registered using a Synergy MX spectrophotometer-spectrofluorometer (BioTek, Winooski, VT, USA). **Pz** solutions were measured in deionized water and ethanol-glycerol mixtures with a percentage of glycerin from 20 to 100% at a **pz** concentration of 5 µM. The absorption spectra were recorded in the wavelength range of 300–700 nm. The fluorescence spectra were registered in the range of 600–850 nm with excitation at 580 nm.

The fluorescence quantum yield was determined relative to rhodamine B (Sigma-Aldrich, Darmstadt, Germany) in water (0.31) [46].

Photobleaching was analyzed in **pz** solutions in the ethanol-glycerol mixture (1:1) in the concentration of 10 μM. In this process, 100 μL of the solution was placed in a well of the 96-well plate at room temperature and irradiated using a LED light source for microplates (λ_ex_ 615–635 nm, 20 mW/cm^2^) [47]. Before and after obtaining different doses of irradiation, the fluorescence spectrum of the **pz** in solution was registered in the range of 590–800 nm with excitation at 570 nm using a Synergy MX microplate reader (BioTek, USA).

### 4.2. Fluorescence Lifetime Registration

The fluorescence lifetime of **pz II** and **pz IV** in ethanol–glycerol solutions was measured according to the previously developed protocol described in [21]. Fluorescence was excited by a SC-450 laser picosecond pulsed source (Fianium Ltd., Southampton, UK) equipped with band-pass interference filters (ChromaTech., Michigan, USA) to select the wavelength range of 580–595 nm. The signal was detected in the spectral range of 640–700 nm by detector connected with time-correlated single-photon counting (TCSPC) system (Becker&Hickl, GmbH, Berlin, Germany). The acquisition time window of the TCSPC system was 0–50 ns, with 1024 channels.

The recorded fluorescence decay curves were deconvoluted using an instrument response function (IRF) recorded as the signal from homogeneous highly scattering non-fluorescent medium. Then, the decay curves were subjected to biexponential fitting, and the intensity-weighted average fluorescence lifetime (*τ*_av_) was calculated using the following equation:

(3) $$\tau_{\mathrm{av}}=\frac{\sum_{i}a_{i}\tau_{i}{}^{2}}{\sum_{i}a_{i}\tau_{i}},$$

where *i* = 1, 2, and *a_i_* and *τ_i_* are the amplitudes and lifetimes of biexponential fitting components for fluorescence decay curve, respectively. The calculations were performed using SPCImage software (Becker&Hickl, GmbH).

To confirm that **pz II** and **pz IV** belong to the group of molecular rotors, we calculated the rate constants of radiative *k_r_* and non-radiative *k_nr_* relaxation of the excited state:

(4) $$\varphi =\frac{k_{r}}{\left(k_{r}+k_{nr}\right)}$$

### 4.3. Cell Lines

Murine glioma GL261, human epidermoid carcinoma A431 and human immortalized keratinocytes HaCaT were cultured at 37 °C under 5% CO_2_ in DMEM (PanEco, Moscow, Russia) containing 2 mM L-glutamine (PanEco, Russia) and 10% fetal bovine serum (FBS, Thermo Fisher, Waltham, MA, USA). The culture medium for glioma GL261 was additionally supplemented with 4.5 g/L glucose (PanEco, Moscow, Russia) and 100 μM sodium pyruvate (Thermo Fisher, USA). At the end of the exponential growth period, the cells were removed with a trypsin-versene solution (1:3) (PanEko, Russia).

### 4.4. Cellular Uptake of Cyanoarylporphyrazines

For the microscopy, cells were seeded in 96-well glass-bottom plates (Corning, NY, USA) at 8 × 10^4^ cells per well and grown overnight. Then, the medium was changed to a serum-free medium containing **pz II** or **pz IV** at a concentration of 10 μM and the cells were incubated for 2 or 4 h. Cells were imaged without changing the medium using an Axio Observer Z1 LSM-710 DUO NLO (Carl Zeiss, Oberkochen, Germany) laser scanning confocal microscope equipped with C-Apochromat 63× water immersion objective lens with numerical aperture 1.2. Fluorescence was excited at 594 nm and registered in the range of 600–670 nm. For semiquantitative analysis, the fluorescence intensity of the cytoplasmic region of the cells was measured using ZEN 2012 program; at least 10 cells in two-three fields of view were analyzed.

For the dynamics study, cells were seeded in glass-bottom 96-well plates (Corning, USA) at a density of 10^4^ cells per well and allowed to attach overnight. Then, the medium was changed to a serum-free medium containing **pz II** or **pz IV** at a concentration of 5 μM, and the fluorescence signal was registered with a Synergy MX plate reader (BioTek, Winooski, VT, USA) during 300 min. Between the measuring time points, the plate with the cells was kept in CO_2_ incubator. Fluorescence was excited at a wavelength of 590 nm and recorded at 650 nm. The registered fluorescence signal was normalized to the maximal value at the end of the incubation to avoid the influence of variations in cell culture density.

### 4.5. Subcellular Distribution of Cyanoarylporphyrazines

The cells were seeded in glass-bottomed 96-well plates (Corning, NY, USA) at a density of 10^4^ cells per well and were grown overnight. Then, the culture medium was changed to a serum-free medium containing **pz II** or **pz IV** at a concentration of 10 μM, and the cells were incubated for 4 h.

For colocalization analysis, the following dyes were added to the medium 30 min before the end of the incubation period according to the manufacturer’s instructions (Thermo Fisher, USA): 0.5 μM LysoTracker green DND-26 for lysosomes, 0.5 μM ER-Tracker for endoplasmic reticulum, 0.5 μM MitoTracker Green FM for mitochondria, 3 μM DAPI for nuclei, and 5 μM BODIPY FL C5-ceramide complexed to BSA for Golgi apparatus. Then, the medium containing pz and the organelle dye was replaced with a fresh serum-free medium.

Cell images were acquired using an Axio Observer Z1 LSM-710 DUO NLO laser scanning microscope (Carl Zeiss, Germany) with an LD C-Apochromat water immersion objective lens 40×/1.1. The following regimens were applied: λ_ex_ 594 nm and λ_em_ 630–670 nm for **pz II** and **pz IV**; λ_ex_ 405 nm and λ_em_ 420–550 nm for DAPI; λ_ex_ 488 nm and λ_em_ 500–550 nm for other organelle dyes.

### 4.6. Analysis of Dark Toxicity and Photodynamic Activity of Porphyrazines

Cells were seeded in a 96-well plate at a density of 6 × 10^3^ cells per well for glioma GL261, 4 × 10^3^ cells per well for carcinoma A431, and 5 × 10^3^ cells per well for human immortalized keratinocytes HaCaT and were grown overnight. Then, the medium was replaced to a serum-free medium containing **pz II** or **pz IV** at concentrations ranging from 0.001 to 70 μM. After 4 h of incubation, the medium was replaced with a porphyrazines-free complete culture medium.

For photodynamic activity estimation, the cells were exposed to light irradiation at the dose of 20 J/cm^2^ using a LED light source (λ_ex_ 615–635 nm, 20 mW/cm^2^) [47]. For dark toxicity estimation, cells loaded with **pz II** or **pz IV** were handled outside of the CO_2_ incubator in the dark for an equal time period. The cells without addition of pz to the medium served as a control.

The MTT test was performed 24 h after cell irradiation; the medium was exchanged with a fresh one with the addition of 0.5 mg/mL MTT-reagent (Alfa Aesar, Lancashire, UK), and the cells were incubated for 4 h. Then, the medium was aspirated, and formed formazan crystals were dissolved in 200 µL of DMSO (PanEco, Russia), and the optical density of the solution was measured at 570 nm using a Synergy MX microplate reader (BioTek, USA). The relative cell viability was calculated as the percentage of mean optical density in the wells with treated cells to the mean optical density in the wells with control cells.

The half-maximal inhibiting concentrations (IC_50_) were calculated from the resulting dose–effect dependences fitted with a four-parameter model for a lognormal distribution using GraphPad Prism (v.6.0) using the equation:

(5) $$V=V_{\min}+\frac{V_{\max}-V}{1+10^{\left(\mathrm{lg}\left({\mathrm{IC}}_{50}\right)-C\right)\cdot \mathrm{SF}}}$$

where Vmax and Vmin are maximal and minimal viability (V) values, respectively; C is the concentration; and SF is the slope factor.

### 4.7. Fluorescence-Lifetime Imaging Microscopy

Cells were seeded in 35 mm glass-bottom Petri dishes (Eppendorf, Hamburg, Germany) at the density of 3 × 10^5^ cells per dish and were grown overnight. Then, the medium was exchanged to a complete culture medium containing **pz II** or **pz IV** at a concentration of 5 μM and incubated for 4 h. Then, the dish with cell culture was placed on a microscope stage without medium replacement; the microscopy experiments were carried out at 37 °C and in an atmosphere of 5% CO_2_.

Images were acquired using an Axio Observer Z1 LSM-710 DUO NLO laser scanning microscopy system with a FLIM module (Becker&Hickl, GmbH, Berlin, Germany) using two-photon excitation at a wavelength of 800 nm and registration of the fluorescence signal in the range of 640–710 nm. At least 0.25 billion photons were collected for every image.

The obtained fluorescence decay curves in every pixel of the images were processed using the SPCImage software (Becker&Hickl, GmbH) and fitted by biexponential decay curve with calculation of the intensity weighted mean lifetime *τ*_av_ using Equation (3).

For photodynamic treatment, ½ of the field of view was exposed to intensive irradiation with a 594 nm laser at a dose of 10 J/cm^2^. FLIM images were acquired before and at different time points after irradiation, and the distribution of τ*_av_* values in irradiated and non-irradiated control areas was analyzed.

## 5. Conclusions

We present two photo-active dyes of the tetracyanotetra(aryl)porphyrazine group, **pz II** and **pz IV**, that combine the properties of potent photosensitizers with viscosity sensors. The compounds have the potential to become beneficial agents for the photodynamic treatment of cancer due to providing the tool for the real-time assessment of tumor cell response directly in the course or immediately after the light irradiation of sensitized tissue. The PDT treatment causes an increase in intracellular viscosity registered by a rise in fluorescence lifetime of **pz II** and **pz IV**. The rise reflects tumor cell impairment and can be used as an objective criterion for light dosimetry.

We anticipate that the low level of dark toxicity and high photodynamic activity are combined with **pz** ability to induce immunogenic cell death, previously shown for two compounds of a similar chemical structure. This property has to be proven in subsequent studies. If this is the case, **pz II** and **pz IV** can be considered as potential photodynamic agents for dosimetry-assisted photodynamic cancer treatment.

## Figures and Tables

**Table 1:** Photophysical properties of **pz II** and **pz IV** in a water solution.

Porphyrazine	λ_abs_, nm	ε	λ_em_, nm	*φ*
**pz II**	594	5.3 × 10^4^	654	0.001
**pz IV**	592	1.8 × 10^4^	650	0.002

λ_abs_, maxima of absorption spectra; λ_em_, maxima of fluorescence spectra; ε, molar extinction coefficient (L × mol^−1^ × cm^−1^); *φ*, quantum yield.

## Data Availability

The data used to support the findings of this study are available from the corresponding author upon request.

## Supplementary Materials

The following are available online, Figure S1: Photobleaching of **pz II** and **pz IV** in ethanol-glycerol solution with 30 cP viscosity. Figure S2: Fluorescence decay curves of pz II and pz IV in ethanol/glycerol mixtures of different viscosity. Figure S3: Dose-dependent photodynamic activity of pz II and pz IV against murine glioma GL261 and human epidermoid carcinoma A431 cells.

## Sample Availability

Samples of the compounds are available from the authors.
