# Supplementary material for: Cyanoarylporphyrazines with High Viscosity Sensitivity: A Step towards Dosimetry-Assisted Photodynamic Cancer Treatment
Source: Molecules. 2021 Sep 25;26(19):5816. doi: 10.3390/molecules26195816 (PMC8510116; doi:10.3390/molecules26195816)
Supplement: Supplementary file 1 [file molecules-26-05816-s001.zip › molecules-1318089-supplementary-done.pdf]

molecules

Supplementary Information

Cyanoarylporphyrazines with High Viscosity Sensitivity: A Step Towards Dosimetry-Assisted Photodynamic Cancer Treatment

Irina V. Balalaeva, Tatiana A. Mishchenko, Victoria D. Turubanova, Nina N. Peskova, Natalia Y. Shilyagina, Vladimir I. Plekhanov, Svetlana A. Lermontova, Larisa G. Klapshina, Maria V. Vedunova and Dmitri V. Krysko

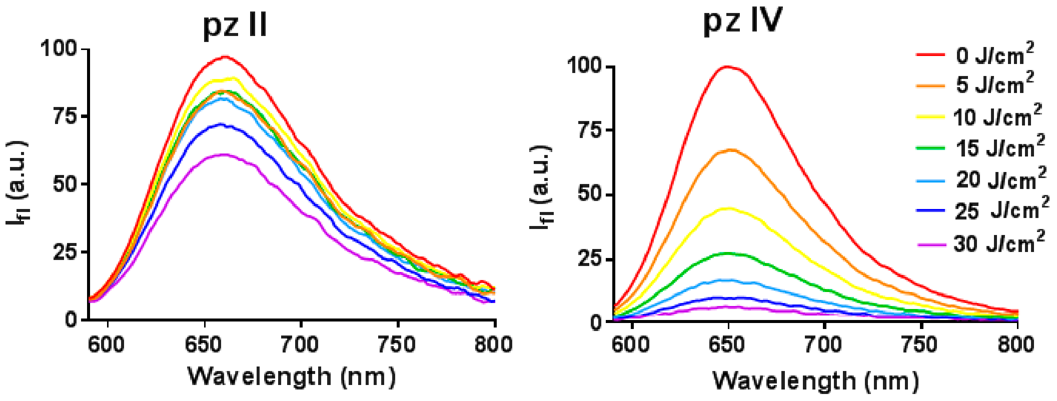

Figure S1. Photobleaching of **pz II** and **pz IV** in ethanol-glycerol solution with 30 cP viscosity induced by irradiation with power density 20 mW/cm^2.

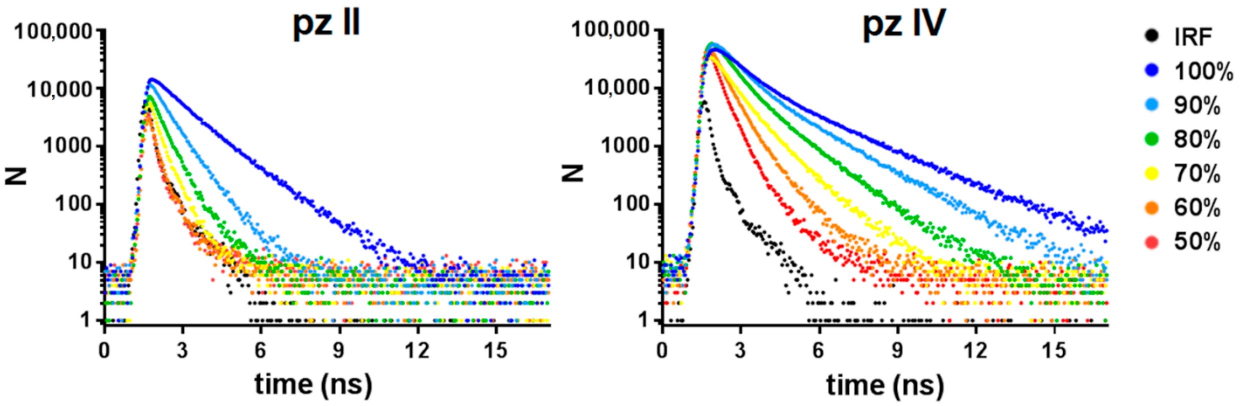

Figure S2. Fluorescence decay curves of **pz II** and **pz IV** in ethanol/glycerol mixtures of different viscosity. The glycerol concentration is indicated. Fluorescence was excited by a SC-450 laser picosecond pulsed source equipped with band-pass interference filters to select the wavelength range of 580-595 nm. The signal was detected in the spectral range of 640-700 nm by detector connected with time-correlated single-photon counting (TCSPC) system. The acquisition time window of the TCSPC system was of 0-50 ns with 1024 channels. The curves were recorded with equal exposition time of 10 ms; thus, the number of registered photons N reflects the relative fluorescence intensity

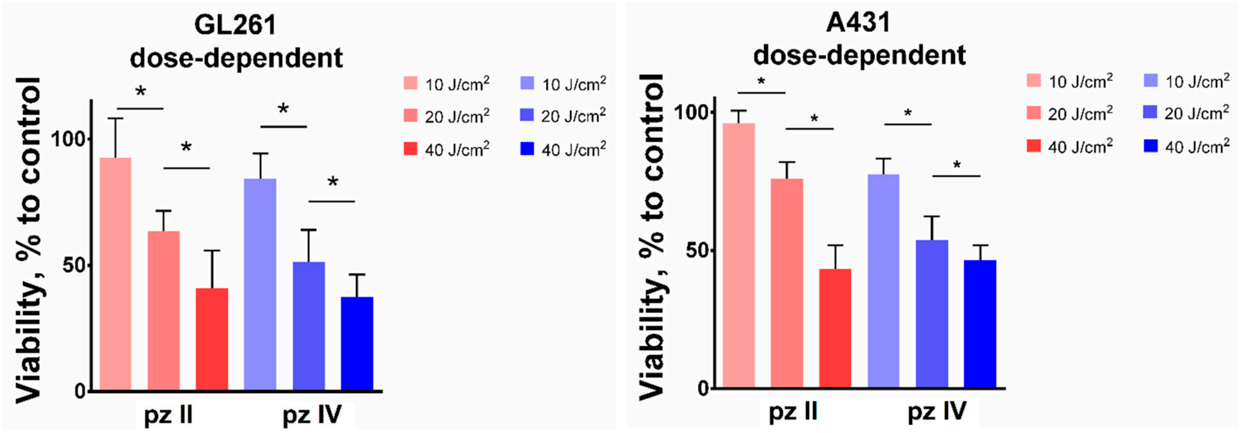

Figure S3. Dose-dependent photodynamic activity of **pz II** and **pz IV** against murine glioma GL261 and human epidermoid carcinoma A431 cells. The cells were incubated with **pz** in serum-free medium for 4 hours and then exposed to light irradiation (λ_{ex} 615-635 nm, 20 mW/cm^2). After 24h, the cell viability was analyzed by MTT assay. The data represent the mean values ± SD (n=3). "*" indicates significant differences between options of treatment (Student's t-test with correction for multiple comparison, $p<0.05$).
